# Narcissistic Traits and Psychological Distress Among Medical Students: Implications for Mental Health Support in Medical Education

**DOI:** 10.3390/healthcare14111504

**Published:** 2026-05-28

**Authors:** Silvana Krnić, Ana Jerončić, Vanessa V. Đogaš, Linda Lušić-Kalcina, Varja Gaić Đogaš

**Affiliations:** 1 Clinical Hospital Center Split, Clinic for Psychiatry, 21000 Split, Croatia; 2 Department of Psychological Medicine, School of Medicine, University of Split, 21000 Split, Croatia; llusic@mefst.hr (L.L.-K.); varja.dogas@mefst.hr (V.G.Đ.); 3 Department of Research in Biomedicine and Health, School of Medicine, University of Split, 21000 Split, Croatia; ana.jeroncic@mefst.hr; 4 Psychiatric Hospital “St. Ivan”, 10000 Zagreb, Croatia; vanessavalentina.dogas@pbsvi.hr

**Keywords:** vulnerable narcissism, grandiose narcissism, psychological distress, medical students

## Abstract

**Highlights:**

**What are the main findings?**

- A substantial proportion of Croatian medical students experience clinically significant psychological distress, with a higher prevalence in early study years and low rates of help-seeking.
- High levels of narcissistic traits—especially vulnerable narcissism—are significantly associated with increased psychological distress and elevated symptoms across multiple domains, particularly interpersonal sensitivity and paranoid ideation.

**What are the implications of the main findings?**

- Early identification of students with elevated vulnerable narcissistic traits may help detect those at higher risk for psychological distress and enable targeted preventive interventions.
- Medical education programs should implement structured mental health support, reduce stigma around help-seeking, and integrate training in emotional regulation, resilience, and interpersonal skills to improve student wellbeing and future patient care.

**Abstract:**

**Background/Objectives:** Growing concern about narcissistic traits in younger generations and their effects on empathy, communication, and mental health underscores the need for focused research on medical students as future healthcare professionals. Medical training carries a substantial psychological burden that can compromise students’ well-being, professional development, and the future quality of patient care. Personality traits, including grandiose and vulnerable narcissistic dimensions, may shape susceptibility to such psychological distress. Therefore, this study aimed to examine narcissistic traits and their associations with psychological distress among Croatian medical students across study years and trait levels. **Methods:** In this cross-sectional study, 413 medical students from the University of Split completed self-administered questionnaires during mandatory classes. Narcissistic traits were assessed using the Pathological Narcissism Inventory (PNI) and psychological distress was measured with the Brief Symptom Inventory (BSI). High pathological narcissistic traits were operationally defined as scores within the highest quartile. Statistical analyses included correlation, comparative, and regression analyses. **Results:** Overall, pathological narcissistic traits were expressed at low to moderate levels and did not differ across academic years. High narcissistic traits were identified in 35% of students, with 15% exhibiting elevated levels of both grandiose and vulnerable dimensions, suggesting an overlap between the two dimensions. Gender differences emerged across both narcissistic dimensions, with women scoring higher on vulnerable and men scoring higher on grandiose narcissism. One-quarter of students met the criteria for clinically significant psychological distress, which was more prevalent in the earlier years of study. High levels of pathological narcissistic traits, particularly vulnerable traits, were significantly associated with clinically significant distress and elevated symptoms across nearly all BSI domains, most notably interpersonal sensitivity and paranoid ideation. Despite this burden, only a minority of distressed students reported seeking psychological help. **Conclusions:** Elevated pathological narcissistic traits are significantly associated with psychological distress in medical students. The high prevalence of distress, combined with low help-seeking rates, underscores the need for early identification strategies and structured mental health support within medical education. Addressing personality-related vulnerability factors may improve student well-being, enhance professional development, and, ultimately, elevate the quality of healthcare delivery.

## 1. Introduction

Contemporary society has undergone substantial cultural and social changes, including shifts in family values, interpersonal norms, and expectations of younger generations. Increased individualization, superficiality, egocentrism, materialism, and shortened attention spans are frequently associated with today’s youth [1,2]. These characteristics are sometimes interpreted as signs of rising narcissistic tendencies, contributing to the concept of “narcissism epidemic” proposed by Twenge et al. [3]. Although the reported rise of narcissism among young people remains controversial [4,5], there is growing interest in understanding how narcissistic traits manifest within specific cohorts of young adults. This is particularly critical in professions where interpersonal sensitivity, ethical judgment, and emotional regulation are essential, such as medicine.

Medical students are exposed to a highly demanding educational environment characterized by heavy academic workload, competitive pressure, and emotionally challenging situations. These factors contribute to high levels of psychological distress and substantial mental health burdens during medical training and are of major concern to both educational institutions and healthcare systems. Previous studies have consistently reported elevated levels of stress, anxiety, depressive symptoms, suicide ideations, and burnout among medical students worldwide, with potentially long-term consequences for professional development, physician well-being, and quality of patient care [6,7,8,9]. Tyssen et al. found that neuroticism, major depression, and negative events predicted suicidal planning [10]. Ionescu et al. demonstrated that in medical education, subclinical psychopathy negatively affects empathy and moral idealism [11]. Consequently, promoting mental health and resilience among medical students has become an important priority in medical education and health policy.

In such high-stress environments, individual psychological characteristics can influence how students respond to the demands of medical training. Personality traits, including narcissistic tendencies, can shape emotional regulation, interpersonal functioning, and coping strategies, showing different resilience levels depending on the type of narcissism. A particularly sensitive issue is the impact of stress as a justification for either grandiose narcissists to systematically abuse their colleagues or for pronounced sensitivity in vulnerable narcissists [12,13]. Narcissistic traits are commonly conceptualized along two related but distinct dimensions: grandiose narcissism, characterized by self-confidence, dominance, and a sense of superiority, and vulnerable narcissism, which is characterized by hypersensitivity to criticism, emotional insecurity, and unstable self-esteem. While certain aspects of narcissism may align with adaptive self-confidence and leadership tendencies, maladaptive forms—particularly those rooted in vulnerability—are linked to greater psychological distress, interpersonal difficulties, and poorer emotional regulation. Evidence suggests that narcissistic traits in medical trainees may negatively affect clinical decision-making, as an inflated sense of self-importance can foster overconfidence and overestimation of one’s abilities, ultimately compromising patient safety and quality of care. According to Yang et al. (2018) [14], narcissists do not learn from the bad outcomes of their decisions; in order to avoid fear and shame, they make decisions driven by arrogance [15] and, as a consequence of these decisions, they react defensively and aggressively [16,17]. Moreover, in highly demanding environments, students with narcissistic traits may exhibit stronger maladaptive tendencies, diminished empathy, and anxiety and depressive symptoms, even in the absence of a narcissistic personality disorder diagnosis. This underscores the importance of early screening and institutional support [18,19,20,21]. Conversely, specific expressions of narcissism, particularly those related to baseline confidence and adaptive self-enhancement, may serve as a buffer against stress and burnout, highlighting the complex nature of narcissistic traits within medical training [22,23]. A deeper understanding of how these psychological factors interact in medical students is essential for developing interventions that foster healthy professional identity formation and high-quality medical practice.

Examining both grandiose and vulnerable narcissistic traits alongside clinically significant psychological distress may provide a deeper understanding of personality-related vulnerability factors within demanding educational environments. Despite growing interest in narcissistic traits and medical education, data from central and eastern European contexts on the topic remain scarce, and no such studies have been conducted in Croatia so far.

Therefore, this study examined grandiose and vulnerable narcissistic traits—as measured by the PNI—alongside clinically significant psychological distress, measured by the BSI, among Croatian medical students. Furthermore, we explored associations between narcissistic traits and clinically significant psychological distress, as well as potential differences in narcissistic traits across genders and academic years. By identifying potential vulnerability factors related to personality and stress, this study aims to contribute to a comprehensive understanding of student well-being and inform institutional interventions designed to strengthen mental health support within medical education and training.

## 2. Materials and Methods

### 2.1. Design

This cross-sectional study included 413 medical students from the University of Split, School of Medicine, Split, Croatia. Data were collected via self-administered questionnaires over two consecutive academic years. The target population comprised all students enrolled in the first, third, and sixth academic years who were present during mandatory class sessions.

### 2.2. Sample Size Estimation

The minimum sample size was calculated based on precision estimates. To achieve a margin of error of ±5% at a 95% confidence level for proportions within the medical student population, a minimum of 381 participants was required, as determined using MedCalc^®^ Statistical Software version 23.0.6 (MedCalc Software Ltd., Ostend, Belgium). This calculation assumed a response distribution of 50%, which provided the most conservative sample estimate. The final study sample exceeded this required number, with 413 students completing the survey.

### 2.3. Data Collection Procedure and Collected Measures

To ensure a high participation rate, paper-based questionnaires were administered to students during mandatory classes. Participants completed the questionnaires individually in the classroom setting. Administration was anonymous, and no personally identifiable information was collected. An investigator was present in the room during data collection to provide standardized instructions, address procedural questions, and ensure independent completion under consistent conditions. Upon completion, participants immediately placed their questionnaires into a sealed ballot box to further guarantee confidentiality.

The survey gathered demographic and background characteristics, including age, gender, academic year, family structure (upbringing in a two-parent household), history of exposure to traumatic events, and previous psychological help-seeking behavior.

Additionally, the survey incorporated two standardized psychological instruments: the Pathological Narcissism Inventory and the Brief Symptom Inventory.

1. PNI (Pathological Narcissism Inventory)

The PNI is a 52-item self-report instrument designed to assess narcissistic traits across two higher-order dimensions: grandiose narcissism and vulnerable narcissism. All items are rated on a 6-point Likert scale, ranging from 0 (not at all like me) to 5 (very much like me). The PNI captures seven sub-dimensions of pathological narcissism, including traits associated with grandiosity (domains: Entitlement Rage, Exploitativeness, Grandiose Fantasy, Self-Sacrificing Self-Enhancement) and vulnerability (domains: Contingent Self-Esteem, Hiding the Self, Devaluing). While the Narcissist Personality Inventory (NPI) has traditionally been used to measure narcissism as a personality trait in the general population, the PNI was developed to capture clinically relevant, pathological aspects, which was our goal.

Despite the inherent limitations of self-reporting measures, the PNI is psychometrically robust, widely utilized in both clinical and research settings, and validated across multiple languages and populations, offering a comprehensive assessment of multifaceted narcissistic pathology. Furthermore, the PNI has been widely used and validated in university settings, often to assess narcissistic traits and their relationship with different psychological and behavioral factors [24,25,26,27,28,29,30]. This study utilized the validated Croatian version of the PNI, which has previously been validated in university student populations [24].

2. BSI (Brief Symptom Inventory)

The BSI questionnaire is a widely used self-report measure of psychological distress and symptoms of psychopathology [31,32,33]. Derived as a shortened version of the Symptoms Checklist-90, it consists of 53 items and typically requires 8–12 min to complete. The BSI can be administered either as a self-report or by an interviewer. It assesses emotional and behavioral functioning across nine symptom dimensions: Somatization, Obsession–compulsion, Interpersonal sensitivity, Depression, Anxiety, Hostility, Phobic anxiety, Paranoid ideation, and Psychoticism. The instrument is suitable for both clinical and non-clinical populations, including medical patients and research participants. Respondents rank each item on a 5-point Likert scale, ranging from 0 (not at all) to 4 (extremely). According to the BSI manual, a T-score greater than or equal to 63 is operationally defined as the cutoff for clinically significant psychological distress [31].

### 2.4. Data Transformation and Statistical Analysis

The PNI yields continuous scores, which were utilized in the primary analyses. Average item scores were calculated separately for the grandiose and vulnerable narcissism dimensions [24,25]. Additionally, because the PNI lacks established clinical cutoff values, an upper-quartile approach was employed to distinguish individuals with relatively elevated narcissistic trait levels (top quartile) from the remainder of the sample. This categorization allowed for the comparison of participants with more pronounced traits and potentially greater psychological vulnerability, a method commonly utilized in personality and clinical psychology research on narcissistic traits [26,27,28,29,30]. Participants scoring within the highest quartile were operationally defined as exhibiting high narcissistic traits. In our sample, this corresponded to a cutoff score of ≥2.49 for vulnerable narcissism (reflecting a response between moderately like me and quite a bit like me) and ≥2.99 for grandiose narcissism (reflecting a response of quite a bit like me).

Statistical analysis was conducted using IBM SPSS Statistics, version 29 (IBM Corp., Armonk, NY, USA).

Descriptive statistics were used to summarize participant characteristics. Categorical variables are presented as frequencies and valid percentages, while continuous variables are reported as means and standard deviations (M ± SD) for normally distributed data or medians and interquartile ranges (Md, IQR) for skewed distributions.

The internal consistency of the PNI domains was assessed using Cronbach’s alpha coefficients. Correlations between continuous psychological measures were evaluated using Pearson’s correlation coefficient (r).

Comparisons between categorical variables, including associations between levels of narcissistic traits (high vs. low/moderate) and psychological distress (yes/no), were analyzed using Pearson’s chi-square test. Adjusted residuals were examined to identify cells contributing most strongly to significant associations. Because continuous psychological measures demonstrated approximately normal distribution, group differences in continuous psychological symptom scores between students with low/moderate and elevated narcissistic traits were assessed using independent-samples Student’s t-tests. Equality of variances was evaluated using Levene’s test prior to t-test interpretation. A one-way analysis of variance (ANOVA) was used to compare mean narcissism scores across academic years.

To examine predictors of clinically significant psychological distress (defined as a BSI score ≥ 63), binary logistic regression analysis was performed, including vulnerable narcissism, grandiose narcissism, age, and gender as independent variables. Model fit and performance were evaluated using the chi-square statistic, Nagelkerke/Cox–Snell coefficients of determination, classification accuracy, and the area under the receiver operating characteristic curve.

Missing data were excluded from individual analyses using available-case analysis. The proportion of missing responses was minimal (≤6% of data) and therefore deemed unlikely to meaningfully bias the results.

Because this study was exploratory and aimed to investigate multiple association patterns rather than test a limited number of predefined confirmatory hypotheses, adjustment for multiple comparisons was not applied. Findings should therefore be interpreted as exploratory and hypothesis-generating.

A *p*-value < 0.05 was considered statistically significant.

## 3. Results

A total of 413 students completed the questionnaires.

### 3.1. Reliability of PNI Domains

Cronbach’s alpha coefficients for the PNI domains indicated acceptable-to-good internal consistency, with values ranging from 0.743 to 0.919. The subscales of Exploitativeness, Self-Sacrificing Self-Enhancement, and Hiding the Self yielded coefficients between 0.74 and 0.77, mirroring findings from the original Croatian validation study [24].

### 3.2. Characteristics of Participants and Psychological Measures

As shown in Table 1, nearly two-thirds of the sample (69.6%) were female, reflecting the gender distribution typical of the medical school population in Croatia. Students were evenly distributed across academic years, with a median age of 21 years (range: 18–29). The majority of participants (82.8%) reported being raised in a two-parent household, and 32.9% indicated that they had experienced at least one traumatic event in their lifetime.

The average PNI item scores for both vulnerable and grandiose narcissism dimensions suggest that narcissistic traits were not highly expressed in this sample. Average scores for vulnerable narcissism fell between “slightly like me” and “moderately like me” responses, whereas grandiose narcissism scores were slightly higher, approximating “moderately like me”. Regarding sociodemographic differences, grandiose narcissism scores did not differ significantly between genders (*p* = 0.755), whereas women exhibited significantly higher vulnerable narcissism scores than men (*p* = 0.006). However, a more detailed analysis of individual PNI domains revealed more nuanced, gender-specific differences within both narcissistic dimensions: men scored significantly higher on Exploitativeness within the grandiose dimension (*p* = 0.021), while women scored higher on Contingent Self-Esteem, Devaluing, and Hiding the Self within the vulnerable dimension (*p* ≤ 0.019). Average PNI scores for both grandiose and vulnerable narcissism remained stable across academic years, with no significant differences observed (*p* ≥ 0.193).

Overall, students reported moderate psychological distress on the BSI. Crucially, one-quarter of the sample (n = 101/25%) met the clinical cutoff for significant psychological distress. Thus, distress was significantly more prevalent among students in their first and third academic years than among final-year students (29–31% vs. 17%, *p* = 0.011). Notably, only 25 (25%) of the distressed students and 41 (14%) of the non-distressed students had previously sought psychological help, representing 17% of the total sample.

### 3.3. High Narcissistic Traits

Following the stratification of participants into low/moderate and high narcissism groups based on PNI quartiles, 130 students (35%) exhibited high levels of either vulnerable or grandiose narcissism. Additionally, 57 students (15%) presented high levels of both vulnerable and grandiose narcissism, indicating a notable overlap between the two dimensions within this cohort (Table 2).

Among students with high levels of both vulnerable and grandiose narcissism, 51 (89.5%) were raised in a two-parent household and 24 (42.1%) reported previous trauma exposure.

Women were overrepresented in this group compared to the overall sample (75.4% vs. 69.5%, *p* = 0.042). This gender discrepancy was likely driven by the occurrence of high vulnerable narcissism, which was significantly more prevalent among women than men (28% vs. 17%, *p* = 0.019); conversely, no significant gender differences were observed for high grandiose narcissism (*p* = 0.702) (Table 3).

### 3.4. Association Between Vulnerable and Grandiose Dimensions

**Figure 1:**
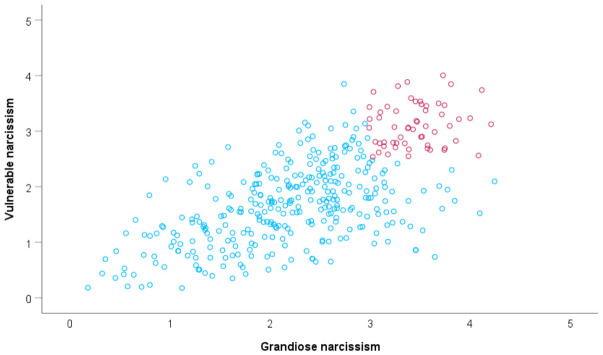
Individual variation in grandiose and vulnerable narcissism presented as a scatter plot of average PNI scores across the two dimensions. Participants in dual high group are marked in red.

Grandiose and vulnerable narcissism scores were moderately correlated in the overall sample (r = 0.65, *p* < 0.001); however, this association was not significant among students exhibiting high levels of both traits (*p* = 0.291; Figure 1).

In this dual high group, the difference between scores was smaller (mean difference ± SD of 0.35 ± 0.46) compared to the remainder of the sample (0.53 ± 0.73), mainly because vulnerable narcissism increased more sharply than grandiose narcissism (Figure 1). This likely explains the weaker correlation between the two traits in this subgroup.

### 3.5. Association Between Psychological Distress and Narcissistic Traits

Higher PNI scores were moderately associated with greater psychological distress (Pearson’s r = 0.49, *p* < 0.001). Logistic regression analysis showed that vulnerable narcissism was a strong positive predictor of clinically significant distress on the BSI (OR = 7.49, *p* < 0.001), whereas grandiose narcissism (OR = 0.42, *p* = 0.001) and older age (OR = 0.87, *p* = 0.049) were negative predictors. Gender was not significantly associated with distress. The overall model was significant, χ^2^(4) = 96.85, *p* < 0.001, explaining 23–34% of the variance and correctly classifying 78.18% of cases (AUC = 0.821). However, despite the performance of this predictive analysis, the cross-sectional nature of the study means that only associations can be inferred.

These findings were further supported when clinically significant psychological distress was analyzed across different levels of narcissistic traits. Higher levels of both vulnerable and grandiose narcissism were associated with greater psychological distress (Table 4). This association was particularly strong for vulnerable narcissism, where students with high trait levels were markedly overrepresented among those reporting clinically significant distress (adjusted residual = 7.0, *p* < 0.001). A similar, though weaker, pattern was observed for grandiose narcissism (adjusted residual = 2.7, *p* = 0.009). More than half of students with high levels of both narcissistic traits (55%) reported elevated psychological distress (adjusted residual = 5.1, *p* < 0.001).

Given these findings, we further examined individual BSI symptom dimensions by comparing average symptom scores between students with low/moderate and high narcissistic traits to identify which psychological symptoms were most strongly associated with elevated narcissism.

As shown in Table 5, students with high grandiose narcissism reported significantly higher scores on almost all BSI symptom dimensions compared with students with low/moderate grandiose narcissism, with the exception of the Somatization dimension. The largest difference was observed for the Paranoid ideation subscale, where students with high grandiose narcissism scored on average 0.44 points higher (representing an 11% increase on a scale from 0 to 4).

Students with high vulnerable narcissism reported significantly higher scores across all BSI symptom dimensions compared with students with low/moderate vulnerable narcissism (Table 6). The largest difference was observed in the Interpersonal Sensitivity subscale, with scores of 0.95 points higher (24% increase), followed by Paranoid ideation and Obsession–compulsion, both showing an average increase of 0.74 points (19% increase).

## 4. Discussion

This study examined the relationship between grandiose and vulnerable narcissistic traits and psychological distress among medical students in Croatia.

Overall, narcissistic traits in our sample were expressed at low-to-moderate levels, with average PNI item scores falling close to the scale midpoint (mean ± SD: 2.36 ± 0.84 for grandiose narcissism and 1.86 ± 0.84 for vulnerable narcissism). These scores are lower than those reported in a previous Croatian study, which found mean scores of 2.85 ± 0.69 for the grandiose and 2.13 ± 0.79 for the vulnerable dimension [24]. However, that previous study utilized a smaller and more heterogeneous sample of university students from various faculties at the University of Zagreb, rather than exclusively medical students. Similarly, studies by Pincus et al. [34] and You (2013) evaluating American and Chinese [27] student populations, respectively, also reported higher baseline scores. Taken together, these findings suggest that narcissistic traits may vary not only across distinct cultural contexts [35] but also according to academic discipline, a notion previously supported by research comparing narcissistic and Dark Triad traits across different college majors [36].

One of the main findings of this study was the strong association between narcissistic traits, particularly vulnerable narcissism, and psychological distress. Students with high vulnerable narcissism scores were significantly more likely to report clinically relevant symptoms across all BSI domains. Indeed, more than half of students with high vulnerable narcissism reported clinically significant distress, compared to only 18% of those with low-to-moderate levels. A similar, though weaker, association was observed for grandiose narcissism (36.3% vs. 22.3%). This is consistent with previous research suggesting that grandiose traits may, in some contexts, be linked to adaptive features such as confidence and perceived competence, while vulnerable narcissism more consistently reflects maladaptive emotional and interpersonal functioning [37]. A more detailed analysis of BSI symptom domains provided a clearer picture of these associations. Students with high vulnerable narcissism scores reported significantly elevated symptoms across all BSI dimensions, with the largest differences observed for interpersonal sensitivity (24% higher), followed by paranoid ideation and obsession–compulsion (both 19% higher). High grandiose narcissism was also associated with elevated symptoms in most domains, with the exception of somatization; the biggest difference was found for paranoid ideation (11% higher compared with students with lower grandiose narcissism scores), followed by interpersonal sensitivity (7% higher). In both groups with highly expressed narcissism, elevated interpersonal sensitivity and paranoid ideation were the most pronounced. These traits may negatively affect teamwork and interpersonal collaboration, both of which are essential for effective medical practice.

Another noteworthy finding was that approximately 15.3% of medical students showed high levels of both grandiose and vulnerable narcissism, which is considerably higher than the 6.25% expected if the two dimensions were completely independent. Female students were significantly overrepresented in this group, likely because they scored higher on vulnerable narcissism, thereby increasing their likelihood of having elevated scores on both dimensions. These findings support contemporary models suggesting that narcissism may fluctuate between grandiose and vulnerable states, rather than representing two completely separate categories [38,39,40]. Specifically, narcissism as a personality trait is conceptualized as a continuous dimension ranging from healthy aspects of self-promotion to pathological expressions that reflect maladaptive personality organization and regulatory mechanisms [41,42,43]. While personality researchers tend to distinguish between dimensions of grandiosity and vulnerability, clinical theorists often emphasize the common features that link these expressions of narcissism [44]. Many contemporary clinical experts in narcissism now recognize that grandiose states self-oscillate or co-occur with vulnerable states of the self and affective dysregulation [25]. Clinical observations also support the “fluctuation” hypothesis [45]. Such fluctuations may contribute to emotional instability and interpersonal difficulties, which are especially relevant in the context of medical education.

Studies suggest that the core structure of narcissism may be similar across genders, while its expression differs [46]. These findings are consistent with our results, which showed a distinct pattern of gender differences. In our sample, gender differences in overall narcissism scores were observed only for vulnerable narcissism, with female students reporting higher scores. However, an analysis of individual domains revealed nuanced gender differences in both narcissistic dimensions, with men scoring higher on the grandiose Exploitativeness domain and women scoring higher on several vulnerable narcissism domains: Contingent Self-Esteem, Devaluing, and Hiding the Self. This pattern closely resembled findings from a previous study of Croatian students using the PNI, which also identified gender differences within both narcissistic dimensions, with men scoring higher on Exploitativeness within the grandiose dimension and female students scoring higher on Contingent Self-Esteem within the vulnerable dimension [24]. These authors also observed that gender-specific effect sizes on PNI scores were small. This suggests that sample characteristics, such as the higher proportion of women in our study (75% vs. 59% in Jaksic), may have masked differences in overall grandiose narcissism scores within our sample. Many studies across different populations and settings have found higher narcissism levels in men than in women, with these differences remaining stable throughout adulthood [47,48,49]. However, Green, who examined gender differences in narcissism using multiple methods and perspectives, suggested that female narcissism may be more strongly associated with vulnerable narcissism, whereas traditional models primarily capture grandiose traits more commonly linked to men [50]. Thus, analyzing narcissism by specific dimensions and domains may help detect subtle differences that are not apparent in overall scores.

Clinically relevant psychological distress was present in a quarter of students and differed significantly across study years. Higher distress levels among first- and third-year students suggest that early stages of medical training are particularly challenging, likely due to academic pressure and adjustment stress. Conversely, lower distress in final-year students may, in part, reflect their greater experience and more effective coping skills. Regardless of the underlying causes, these findings highlight the need for targeted psychological support early in the curriculum.

It is noteworthy that only 17% students in total sought psychological help: only 25% of those experiencing clinically significant distress and 14% of those who were not distressed at the time of the survey. The finding aligns with previous research showing that although approximately 20% of college students experience mental health problems, only one-sixth of them receive minimally adequate treatment, with treatment rates ranging from 6% in low-income countries to 23% in high-income countries [51]. While the limited availability of appropriate mental health services may contribute to these low rates of help-seeking, additional barriers include stigma, low motivation to seek help, lack of time, and normalization of stress as an expected part of student life [52]. A recent scoping review of 33 studies reported that the most frequently identified barriers were the fear of a negative effect on residency or career opportunities, fear of confidentiality breaches, stigma and the fear of shaming from peers, a lack of perceived seriousness or the normalization of symptoms, a lack of time, and the fear of documentation on the academic record [53].

Taken together, these studies suggest that many barriers to mental healthcare among medical students stem from fears of academic or professional repercussions, as well as confidentiality concerns. Despite growing awareness and ongoing efforts to reduce the stigma surrounding mental illness, many medical students continue to struggle with seeking appropriate psychological support. Consequently, future student support programs must address these specific challenges. Key recommendations include establishing counseling services independent of faculty administrative structures and enabling anonymous, self-referral pathways to psychological care. Notably, online support has been shown to mitigate the barrier of shame, which is particularly pronounced in individuals with vulnerable narcissism. Furthermore, targeted anti-stigma campaigns should be launched within the academic community to emphasize that seeking psychological support is a sign of professional responsibility, rather than weakness.

### 4.1. Limitations

This study has several limitations. First, its cross-sectional design does not allow for conclusions regarding causality between narcissistic traits and psychological distress; thus, the observed associations should be interpreted with caution. Second, the use of self-report instruments may introduce social desirability or response bias, particularly given that individuals with elevated narcissistic traits may overestimate or underestimate their psychological functioning. Nonetheless, despite these inherent limitations of self-report measures, the PNI is a well-validated and widely used instrument for assessing narcissistic pathology in the student population. Third, because this study was conducted at a single institution, the generalizability of the findings to other cultural or educational contexts may be limited. However, our reliability analysis of the PNI domains closely mirrors findings from a Croatian validation study conducted among students at a different university center [24]. This suggests that while overall narcissism scores were lower in our sample, the underlying psychometric patterns remained well preserved. These lower scores may be related to the specific characteristics of medical students, as also suggested by the literature and our unpublished comparative data across different faculties. Finally, although the use of the upper quartile to define high narcissistic traits was methodologically justified, it does not represent a clinical threshold and remains sample dependent. Nonetheless, this approach enabled us to identify distinct association patterns between participants with elevated narcissistic traits and those with lower scores, supporting its exploratory and discriminative value in the present study.

### 4.2. Clinical and Educational Implications

This study contributes to the growing body of literature by providing insight into the relationship between narcissistic traits and psychological distress within a central and eastern European medical student population. Furthermore, it highlights the importance of considering personality-related vulnerability factors to better understand student well-being.

From a practical perspective, these findings support the need for structured mental health strategies within medical education. Early identification of at-risk students, particularly those with elevated vulnerable narcissistic traits, may facilitate timely interventions. Medical schools could benefit from implementing screening programs, mentoring systems, and accessible, confidential counseling services. In addition, interventions aimed at improving emotional regulation, resilience, and interpersonal skills may help support students experiencing elevated stress levels, thereby enhancing their well-being and future professional functioning.

## 5. Conclusions

This study provides new insights into the relationship between narcissistic traits and psychological distress among medical students. Although overall narcissism levels in our sample were lower than those reported in previous studies, higher levels of both grandiose and vulnerable traits moderately correlate with clinically significant distress. While both narcissistic dimensions highlighted interpersonal sensitivity and paranoid ideation as key symptoms, vulnerable narcissism exhibits broader, stronger associations across all psychological symptom domains.

A notable subgroup of students exhibited simultaneously high levels of grandiose and vulnerable narcissism, underscoring the growing evidence that these dimensions can co-occur and fluctuate within individuals.

Our findings also support the increasingly accepted view that narcissism tends to be more grandiose in men and more vulnerable in women; these gender differences were subtle and became visible only when specific narcissism dimensions and domains were analyzed. Consequently, this insight may help explain the inconsistent findings in previous research.

The present findings carry important implications for medical education and institutional student support systems. The high prevalence of clinically significant psychological distress—particularly among students in the earlier years of training—underscores the need for systematic and proactive mental health strategies within medical curricula, especially considering the difficulties found in interpersonal sensitivity and paranoid ideation. The strong association between vulnerable narcissistic traits and distress suggests that certain personality-related vulnerability patterns may increase sensitivity to academic stress, interpersonal challenges, and perceived failure. Early identification of students at risk, through structured well-being screening programs or confidential mental health assessments, may help prevent escalation of symptoms. Furthermore, integrating structured mentoring programs, resilience training, and workshops focused on emotional regulation, reflective practice, and interpersonal communication could mitigate maladaptive responses to stress.

Although one quarter of the students met the criteria for clinically significant distress, only 17% sought psychological help. Given this observed gap between psychological distress and help-seeking behavior, medical schools should strengthen mental health support within medical education by prioritizing early detection, reducing stigma, enhancing access to care, and encouraging adaptive coping strategies. Practical implications for early detection include early screening of students for psychological distress and the introduction of structured mentoring systems. The high correlation between grandiose and vulnerable narcissism suggests that professionals in student counseling centers should not merely look for “typical grandiose narcissists”. Instead, they must be aware that behind the mask of self-confidence often lies a deep emotional fragility that easily turns into distress. Furthermore, student counseling centers must operate independently of faculty administrative structures to enable anonymous, self-referral access to psychological support. Ensuring that the utilization of mental health services is not documented in academic records may help reduce fears of academic or professional repercussions, as well as confidentiality concerns, both of which are recognized in the literature as key barriers to help-seeking.

Addressing these factors during undergraduate training may not only enhance student well-being but also foster healthier professional identity formation, improved teamwork, and, ultimately, higher-quality patient care within future healthcare systems.

## Figures and Tables

**Table 1:** Demographic, psychosocial, and psychological characteristics of participants; N = 413.

Characteristics		Statistics
Socio-demographic characteristics		
Age [years], Md (IQR)		21 (20–24)
Female, N (%)		287 (69.5)
Year of study, N (%)	1st	142 (34.4)
	3rd	128 (31.0)
	6th	143 (34.6)
Psychosocial characteristics		
Family with both parents, N (%)		339 (82.1)
Exposure to traumatic event, N (%)		134 (32.9)
Previous psychological help, N (%)		70 (17.2)
Psychological characteristics		
Average PNI item score *, M ± SD (range)		
vulnerable narcissism		1.86 ± 0.84 (range 0.18–4.0)
grandiose narcissism		2.36 ± 0.84 (range 0.18–4.2)
BSI score, Md (IQR)		42 (24–64)

Md—median, IQR—interquartile range, M—mean, SD—standard deviation, PNI—Pathological Narcissism Inventory, BSI—Brief Symptom Inventory. Shown are valid percentages. * Item scores ranged from 0 to 5—from 0 (not at all like me) to 5 (very much like me).

**Table 2:** Distribution of students by levels of vulnerable and grandiose narcissism.

	Vulnerable Narcissism	
Grandiose Narcissism	Low/Moderate, N (%)	High *, N (%)
Low/Moderate, N (%)	243 (65.1)	36 (9.7)
High, N (%)	37 (9.9)	57 (15.3)

Values represent valid total percentages. * High narcissistic traits were operationally defined as scores within the highest quartile of the respective PNI dimension; all remaining participants were classified as having low/moderate narcissistic traits.

**Table 3:** Association between gender and levels of narcissism.

Level of Narcissism	Narcissistic Dimensions	Women, N (%)	Men, N (%)	*p*-Value
Low/moderate	Vulnerable	188 (71.8)	96 (83.5)	0.019
High *		74 (28.2)	19 (16.5)	
Low/moderate	Grandiose	209 (76.3)	89 (74.2)	0.702
High		65 (23.7)	31 (25.8)	

Values represent valid total percentages. * High narcissistic traits were operationally defined as scores within the highest quartile of the respective PNI dimension; all remaining participants were classified as having low/moderate narcissistic traits.

**Table 4:** The association between psychological distress and levels of narcissism.

Psychological Distress *	Narcissistic Dimensions	Level of Narcissism, N (%)		
		Low/Moderate	High **	*p*-Value
No	Vulnerable	230 (82.4)	40 (44.9)	<0.001
Yes		49 (17.6)	49 (55.1)	
No	Grandiose	227 (77.7)	58 (63.7)	0.009
Yes		65 (22.3)	33 (36.3)	

Values represent valid percentages. * Psychological distress is defined as BSI score ≥ 63. ** High narcissistic traits were operationally defined as scores within the highest quartile of the respective PNI dimension; all remaining participants were classified as having low/moderate narcissistic traits.

**Table 5:** Average item scores on BSI dimensions by level of narcissistic grandiosity.

	Level of Grandiose Narcissism		*p*-Value
	Low/Moderate, M ± SD	High, M ± SD	
Somatization	0.74 ± 0.65	0.87 ± 0.76	0.138
Obsession–compulsion	1.24 ± 0.82	1.52 ± 0.86	0.005
Interpersonal sensitivity	0.95 ± 0.85	1.24 ± 1.02	0.012
Depression	0.9 ± 0.76	1.12 ± 0.87	0.022
Anxiety	1.03 ± 0.73	1.24 ± 0.83	0.020
Hostility	0.81 ± 0.68	1.03 ± 0.83	0.019
Phobic anxiety	0.53 ± 0.64	0.75 ± 0.81	0.016
Paranoid ideations	0.78 ± 0.63	1.22 ± 0.85	<0.001
Psychoticism	0.61 ± 0.64	0.8 ± 0.75	0.025

**Table 6:** Average item scores on BSI dimensions by level of narcissistic vulnerability.

	Level of Vulnerable Narcissism		*p*-Value
	Low/Moderate, M ± SD	High, M ± SD	
Somatization	0.69 ± 0.62	1.03 ± 0.82	<0.001
Obsession–compulsion	1.12 ± 0.74	1.86 ± 0.89	<0.001
Interpersonal sensitivity	0.79 ± 0.77	1.74 ± 0.98	<0.001
Depression	0.79 ± 0.72	1.48 ± 0.85	<0.001
Anxiety	0.93 ± 0.68	1.52 ± 0.84	<0.001
Hostility	0.74 ± 0.66	1.25 ± 0.8	<0.001
Phobic anxiety	0.48 ± 0.62	0.95 ± 0.84	<0.001
Paranoid ideations	0.71 ± 0.6	1.45 ± 0.8	<0.001
Psychoticism	0.52 ± 0.6	1.08 ± 0.76	<0.001

## Data Availability

The datasets generated and analyzed during the current study are not publicly available due to privacy and ethical restrictions but are available from the corresponding author upon reasonable request.

## Abbreviations

The following abbreviations are used in this manuscript:

PNI	Pathological Narcissism Inventory
BSI	Brief Symptom Inventory
